# The Development of a Cost-Effective Infant Intraosseous Infusion Simulator for Neonatal Resuscitation Program Training

**DOI:** 10.7759/cureus.18824

**Published:** 2021-10-16

**Authors:** Julia Micallef, Artur Arutiunian, Jennifer Hiley, Andy Benson, Adam Dubrowski

**Affiliations:** 1 Health Sciences, Ontario Tech University, Oshawa, CAN; 2 Neonatal Resuscitation, Lakeridge Health Hospital, Oshawa, CAN; 3 Central East Prehospital Care Program, Lakeridge Health Hospital, Oshawa, CAN

**Keywords:** 3d printing, training, simulator, intraosseous infusion, simulation-based medical education

## Abstract

Simulation-based medical education (SBME) employs realistic simulators to allow physicians and medical students to learn and practice high acuity, low occurrence (HALO) skills such as the intraosseous (IO) infusion. Previous research was done to develop and evaluate a three-dimensional (3D)-printed adult proximal tibia IO simulator and was rated as a valuable and realistic medical education training tool. This report focuses on implementing this IO simulator for neonatal resuscitation program (NRP) training purposes, as well as to explain the process of redeveloping the previous adult IO simulator and the development of a stand, called the maxSIMbox, to hold the simulators, as well as the tools needed to perform an IO infusion. The feedback provided from stakeholders was helpful, with an emphasis on providing stability to both the infant IO simulator and the maxSIMbox. From this feedback, a functional and cost-effective simulator was developed to practice this HALO skill and is currently being used for NRP training.

## Introduction

Simulation-based medical education (SBME) is a rapidly growing field that employs realistic simulators to allow physicians and medical students to practice clinical procedures without causing unnecessary patient harm [1]. The introduction of three-dimensional (3D) printing into medical education presents a low-cost alternative to expensive simulators for practicing procedural skills [2]. Specialties such as emergency medicine, especially in rural communities, can benefit from SBME by allowing learners and professionals to learn and practice rare but potentially life-saving skills in a controlled and safe environment. One such high acuity, low occurrence (HALO) skill is the intraosseous (IO) infusion.

We have recently published a report describing the development and evaluation of a 3D-printed adult proximal tibia IO simulator [3]. This initial report intended to assess the realism and obtain suggestions from content experts to improve the simulator and produce a useful teaching tool. The target population of intended users was sampled from the 2019 Academic & Wellness Resident Workshop held at the Bay Roberts Hotel and Tilt Room on November 23, 2019 (Newfoundland and Labrador, Canada). The workshop included a procedural and trauma simulation session where residents were offered the opportunity to practice specialized procedures required by rural emergency department (ED) physicians. Two adult proximal tibia IO simulators were set up as a simulation station for this session. This initial data collection was later supplemented by a second data collection session taking place at the Carbonear General Hospital ED in Carbonear (Newfoundland and Labrador, Canada). ED physicians and registered nurses (RNs) were allowed to test the simulator. In total, 15 residents (12 from postgraduate year one and three from year two), six ED physicians, six RNs, and one unspecified practitioner participated in the study. Overall, the IO simulator was rated as a valuable and useful medical education training tool. Both groups made several suggestions to improve the realism of the simulator [3]. The simulator was developed and manufactured based on the “design-to-value” approach [4], where the clinicians and the designers seek to build the best possible product (i.e. simulator) without much regard for the cost. This approach puts a constraint on realism and allows costs of design and manufacturing to vary.

More recently, we attempted to implement this IO simulator for neonatal resuscitation program (NRP) training in an urban simulation training center at Lakeridge Health Education and Research Network (LHEARN, Oshawa, Ontario, Canada). In addition to the size differences between the two simulators (i.e. the adult and the infant), because of the high volume of training and needs for disposable simulators (coronavirus disease 2019 [COVID-19] related), this infant version of the simulator was to be designed based on the “design-to-cost” approach. That is, the cost was a consideration from the start of the design process with requirement design to reduce the costs. More specifically, the design and manufacturing aimed to be as lightweight and as efficient as possible to reduce the costs, while sacrificing some of the realism of the final product.

Given the polarity of the contexts and plans for subsequent and rapid implementation of the new infant IO simulator, we used elements of the Consolidated Framework for Implementation Research (CFIR) [5,6] to look at contextual factors that may help with future implementation.

The purposes of this technical report are threefold. The first is to describe the process of redeveloping the initial IO simulator using adult tibial bones as a starting point [3]. The second is to describe the new, infant and parsimonious IO simulator. The third is to provide the initial evaluation of the simulator, with early exploration of factors leading to better implementation, as a training tool by simulation educators in the fields of paramedicine and NRP training.

## Technical report

This report is described using a modified context, input, process, and product (CIPP) simulator to optimize applicability to various learning environments, educational context, inputs, processes, and expected outcomes [7].

Context

The implementation for the infant IO simulator was an urban training hospital located at the Lakeridge Health Education and Research Network (LHEARN, Oshawa, Ontario, Canada). The host program was the NRP which provides skills maintenance programs periodically to the NRP teams in the hospital. Paramedics who are trained in similar skills through the Central East Prehospital Care Program (CEPCP), Lakeridge Hospital (Oshawa, Ontario, Canada) also expressed interest in utilizing the IO simulator after the redesign and therefore were considered a group of potential stakeholders and potential users.

There were a total of four meetings with the stakeholders, each lasting approximately 30 minutes. Of the four meetings, three were with the NRP team at Lakeridge Hospital in the LHEARN center, and one was with the paramedics of the CEPCP. During each meeting, the stakeholders would individually perform the IO infusion on the simulator at least three times before providing verbal feedback for the redesign.

We have followed the first phase of the Adapted Implementation Model for Simulation (AIM-SIM) process described earlier [8], which includes three implementation phases: (a) stakeholder engagement and context exploration; (b) pre-implementation planning; and (c) implementation with monitoring and ongoing evaluation. During the initial stakeholder meeting, three re-design constraints were articulated. First, the re-design simulator needs to be more cost-effective, with a total cost under $25 (CAD) and operation costs under $1 (CAD) per user. Second, because of the nature of the training and storage issues between the training, the new IO simulator needs to be self-contained for all the necessary replacement parts. Third, the re-designed IO simulator will create an easy platform to provide training opportunities to four trainees at a time, while adhering to COVID-19 imposed safety protocols, such as not sharing any parts of the simulator and enabling quick and effective disinfection between uses. The final number (in grams) of materials used, costs, and the 3D printer parameters to make one IO simulator can be seen in Table 1.

**Table 1:** Summary of materials used, costs, and 3D printer parameters. CAD, Canadian dollar; IO, intraosseous; 3D, three-dimensional.

Part	Material quantity used (g)	Multiples	Total material (g)	Costs ($ in CAD)	Infill (%)	Wall thickness (mm)
maxSIMbox (stand)	454	1	454	15.89	20	1.2
Table attachment	66	1	66	2.31	20	1.2
IO slide	75	4	300	10.5	20	1.2
IO bones (infant)	4	8	32	1.12	0	1.2
Tools slide	90	1	90	3.15	20	1.2
Suction cups	1	4	4	5.04	N/A	N/A
TOTAL			946	38.01		

Inputs

The infant IO simulator was designed by an undergraduate life sciences student (Ontario Tech University, Oshawa, Ontario, Canada) with a background in 3D printing. To ensure effective feedback and communications with the NRP and CEPCP training teams, the student was physically co-located with these teams within the hospital. Therefore, the development followed a concept of a living lab, which is a user-centered, open-innovation ecosystem integrating concurrent research and innovation processes within a partnership [9]. There were two neonatal resuscitation nurses and three paramedics simulation experts from Lakeridge Health Hospital who provided continuous feedback. This research and the partnership are part of a local simulation research and development network (maxSIMhealth.com).

**Figure 1:**
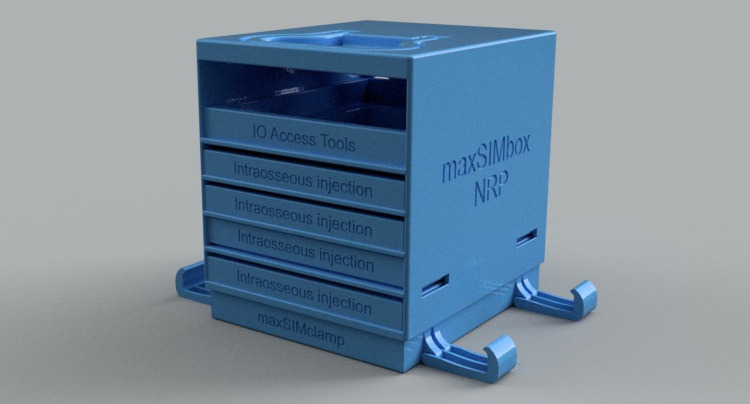
The 3D-rendered version of the maxSIMbox. 3D, three-dimensional.

We used the previously published digital assets [3] for the redevelopment. The files were manipulated as a stereolithography (.stl) file in Fusion360™ (Autodesk Inc., San Rafael, CA). Using a secure digital (SD) card, the 3D-rendered files were transferred to an Ultimaker S5 3D printer (Ultimaker B.V., Utrecht, Netherlands) and were printed using white Ecotough™ polylactic acid (PLA) filament material (Mississauga, Ontario). A stand (referred to as maxSIMbox) for the separate tibial bone stimulators was designed using Fusion360™ so that the bones could friction-fit into the base (Figure 1). As depicted in Figure 1, the maxSIMbox was designed to hold five slides, four of which could hold two tibial head bone fragments each and one slide for placing either spare parts or clinical tools; the box was attached to a clamp with suction cups to provide stability. The clamps were designed with a dual purpose: to serve as holders for the maxSIMbox, as well as holding the slides when in use (Figure 1). The stand .stl file was also transferred to an Ultimaker S5 3D printer and printed using Ecotough™ PLA filament material.

Process

Adhering to the first phase of the AIM-SIM, we held a pre-implementation stakeholder meeting, and utilized a subset of CFIR constructs dealing with the domain of “Intervention Characteristics,” and the following points were used to guide the re-development:

1. Relative advantage: Stakeholders’ perception of the advantage of implementing the intervention versus an alternative solution. The current best practices involve using a solid foam tibial bone stimulator (Sawbones, Vashon Island, Washington). The relative advantage would be achieved through a better cost-to-use ratio. Storage and ease of cleaning were also identified as contributing design factors.

2. Complexity: The perceived difficulty of the intervention, reflected by duration, scope, radicalness, disruptiveness, centrality, and intricacy, and the number of steps required to implement. The design team focused on “lean” design that minimized the number of components, specifically replicable comportments, as well as storage when not used. For example, a decision was made to eliminate the skin layer to reduce the cost and complexity.

3. Design quality: Perceived excellence in how the intervention is bundled, presented, and assembled. The design team decided to develop “maxSIMbox” (Figure 1) to package all components for ease of storage and use.

4. Intervention source: Perception of key stakeholders about whether the intervention is externally or internally developed. The endpoint users were involved in all aspects of design and manufacturing to address this factor.

The stakeholders provided verbal feedback outlining any changes to the digital and physical iterations of the simulator. The feedback included two main categories: tibial bones and maxSIMbox. For the tibial bones, the feedback was (1) securing the simulators to the plate with a custom groove to minimize movement during drilling, (2) reduction of the thickness of the bone, and (3) reducing the size of the bones and the target segment (i.e. the head of the tibia only). For the maxSIMbox, the feedback was (1) inclusion of suction cups to hold the simulator, (2) adding a groove to prevent the bones from moving, (3) reducing the size of the slides to accommodate four separate sets of simulators (two simulators per slide), and (4) extending the height of the maxSIMbox to accommodate for spare parts and possibly equipment such as the Arrow® EZ-IO® Power Driver (Teleflex Medical, Research Triangle Park, NC, USA), EZ-IO® needle set, or custom 3D-printed drill bit made to fit EZ-IO® needle that can be operated using a conventional drill.

**Figure 2:**
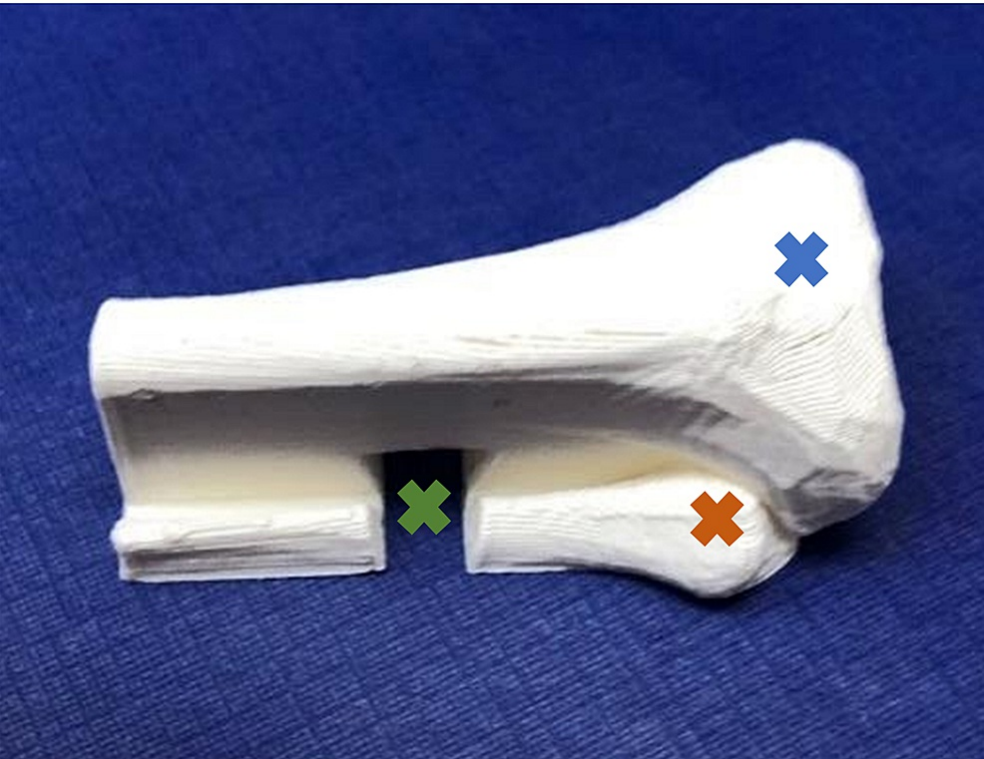
Infant IO bone after feedback. Blue X: head of tibia; orange X: head of fibula; green X: groove; IO: intraosseous.

**Figure 3:**
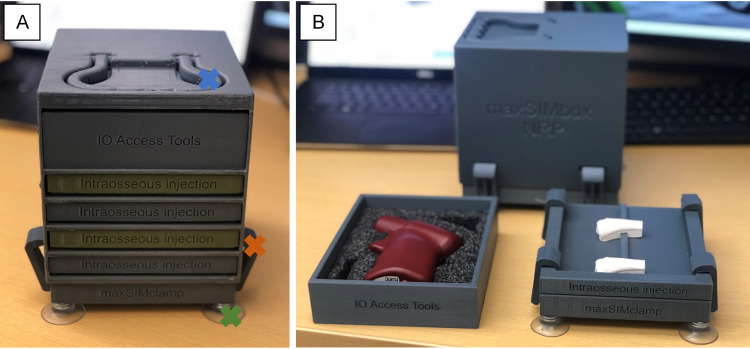
‘A’ shows the maxSIMbox and its adjusted components, while ‘B’ depicts how the clamps are used to secure the slide holding the IO bones (held in the slide via friction-fit) shown on the right as well as the slide holding the IO access tools shown on the left. Blue X: handle; orange X: clamp; green X: suction cup; IO: intraosseous.

After the feedback was received, the tibial bone fragments and maxSIMbox were adjusted in Fusion 360, shown in Figure 2 and Figure 3, respectively. The tibial bones were 3D printed with white PLA plastic material at 0.2 layer height and 2.0 mm wall thickness and hollow on the inside. It takes approximately 20 minutes to print one tibial bone fragment, and a total of three days to print the maxSIMbox.

Products

Table 1 shows the costs and manufacturing requirements of the final NRP simulator. All elements included in the box were deemed acceptable by the stakeholders and are currently used routinely in NRP training. We have developed a process whereby the NRP lead requests replacement parts immediately after the training, which are manufactured in the laboratory and stored with the maxSIMbox to be ready for subsequent training sessions.

## Discussion

This report aimed to redevelop the initial IO simulator using adult tibial bones [3] for infants, provide a description of the new IO simulator for infant tibia bones, and provide the initial evaluation of the simulator as a training tool by simulation educators in the fields of paramedicine and NRP training. The feedback provided from stakeholders was helpful, with an emphasis on providing stability to both the infant IO simulator and the maxSIMbox. From this feedback, a functional and cost-effective simulator was developed to practice the HALO skill, IO infusion via SBME.

In addition to providing a description, design, and manufacturing specification of the infant IO simulator, the current report advances our understanding and methodologies underpinning collaborative design in healthcare training and simulation in two ways.

First, to the best of our knowledge, this is the only report that describes “design-to-cost” development as opposed to the “design-to-value” approach. Many in the field of healthcare simulation consider realism (aka fidelity) the most important design feature [10], with most designs being based on the “design-to-value” approach [4], where the simulators are built without much regard for the cost. However, based on several position papers and evidence gathered, we know that this is not always the case and realism is distinctly different from the simulators’ features that support skills development [11-13]. For this reason, the current simulator was to be designed based on the “design-to-cost” approach. That is, the cost was a consideration from the start of the design process with requirement design to reduce the costs, sacrificing some of the realism of the final product while maintaining educational values [4]. This technical report, and the simulator, are in the early development and piloting phase of research and development. Specifically, the design, validation, and test of efficacy, and subsequent implementation will follow a previously described framework for the development of complex interventions [13]. According to this framework, this technical report is designed to illustrate the design and modeling of our intervention, which is the development of the IO trainer. Piloting work, validation, and definite evaluation of the efficacy are planned for the future and outside of the scope of the current work. However, because we included an expert panel to design the simulator, this report contains elements of the content validity of the simulator.

Second, to the best of our knowledge, this is the only report that utilizes frameworks derived from implementation science to guide the development. More specifically, before the design cycle, we were interested to learn from the experts and potential endpoint users of the simulator what factors would optimize the future implementation of the simulator in the real-world setting. In light of the COVID-19-related constraints, this step drastically changed the initial and final design of the simulator. Specifically, the research and design team conceived bone fragments with replaceable parts to optimize costs (i.e. similar to the original design [3]) and the expert panel provided feedback guided by the factors known to optimize implementation success. The feedback included the need for the simulator to be embedded within a portable unit (maxSIMbox), which also carries the instruments. The simulator sacrifices some features like skin; and the simulator can be cleaned, replaced, and stored with ease. This feedback would not be possible without early engagement with the stakeholders, highlighting the importance of an iterative approach to research and development described in our earlier work [7].

Previous trials which tested the effectiveness of SBME found that SBME can improve patient safety and self-confidence to perform the skill [14]. This means that SBME can be utilized in a clinical context for emergency responders and those involved in neonatal resuscitation who come across HALO skills that have not been regularly practiced. This is especially true for the emergency responders in rural areas who experience HALO procedures, such as IO infusions.

## Conclusions

This technical report contributes to the growing body of literature on the use of additive manufacturing in simulation in three significant ways. First, it provides a technical description of a parsimonious (optimizing costs and educational function) infant IO simulator system. Second, it demonstrates the use of the “design-to-cost” approach, which is more in line with the current conceptualization of the role of realism (aka fidelity) in simulation-based education and training. Third, it highlights the importance of using an end-point user-based design, which in our case was based on a subset of constructs and factors known to optimize future implementation.
